# The primary cilium protein folliculin is part of the autophagy signaling pathway to regulate epithelial cell size in response to fluid flow

**DOI:** 10.15698/cst2019.03.180

**Published:** 2019-02-25

**Authors:** Naïma Zemirli, Asma Boukhalfa, Nicolas Dupont, Joëlle Botti, Patrice Codogno, Etienne Morel

**Affiliations:** 1 Institut Necker-Enfants Malades (INEM), INSERM U1151-CNRS UMR 8253.; 2 Université Paris Descartes-Sorbonne Paris Cité, F-75993, Paris, France.; 3 Université Paris Denis Diderot Sorbonne Paris Cité, F-75993, Paris, France.

**Keywords:** fluid flow, shear stress, autophagy, folliculin, autophagy, primary cilium

## Abstract

Autophagy is a conserved molecular pathway directly involved in the degradation and recycling of intracellular components. Autophagy is associated with a response to stress situations, such as nutrients deficit, chemical toxicity, mechanical stress or microbial host defense. We have recently shown that primary cilium-dependent autophagy is important to control kidney epithelial cell size in response to fluid flow induced shear stress. Here we show that the ciliary protein folliculin (FLCN) actively participates to the signaling cascade leading to the stimulation of fluid flow-dependent autophagy upstream of the cell size regulation in HK2 kidney epithelial cells. The knockdown of FLCN induces a shortening of the primary cilium, inhibits the activation of AMPK and the recruitment of the autophagy protein ATG16L1 at the primary cilium. Altogether, our results suggest that FLCN is essential in the dialog between autophagy and the primary cilium in epithelial cells to integrate shear stress-dependent signaling.

## INTRODUCTION

Autophagy is an evolutionary conserved stress-response process by which cells break down intracellular components, damaged organelles and proteins aggregates or pathogens, to ensure cellular quality control and homeostasis. It is induced in response to various stress types such as nutrient deprivation, cytotoxic agents, and hypoxia. Autophagy involves the sequestration of cytoplasmic material in a double membrane organelle named autophagosome, which subsequently fuses with the lysosome to degrade and recycle autophagosomal cargoes [1]. We have recently shown that in kidney epithelial cells (KECs) autophagy is induced in response to fluid flow-provoked shear stress and that this fluid flow-dependent autophagy regulates cell volume [2]. We have demonstrated that shear stressinduced autophagy is triggered by a signaling cascade emanating from the primary cilium located at the apical side of epithelial cells [2]. The primary cilium, which is composed of a basal body and an axoneme, is a microtubulebased organelle present at the surface of various cell types [3] and plays a critical role in maintaining tissue homeostasis by sensing extracellular mechanical and chemical stimuli [4].

To better understand the molecular mechanisms of fluid flow-induced autophagic response and cell volume regulation, it is important to identify additional players located at the primary cilium. The folliculin protein (FLCN) presents interesting features in that respect. A pool of FLCN is located at the primary cilium [5] and regulates the AMPK/mTOR signaling pathway in response to fluid flow [6]. Indeed, FLCN promotes the recruitment of LKB1 kinase to basal bodies where it activates AMPK, which in turn inhibits mTOR activation [6]. FLCN has moreover been associated with autophagy pathway regulation [7–9].

FLCN is a conserved tumor suppressor protein expressed in most cell types. Loss-of-function mutations of the FLCN gene are associated with the autosomal dominant disorder Birt-Hogg-Dubé (BHD) syndrome. It is characterized by benign hair follicle tumors, pneumothorax, cysts and renal cancer occurrence [10], and has an estimated prevalence of 1/200.000. Importantly, considering the abnormal ciliogenesis and canonical Wnt signaling, the BHD syndrome is also considered as a ciliopathy [5]. FLCN can form a complex with its two main partners FNIP1 and FNIP2 (Folliculin interacting protein 1/2). Different studies suggest a role of the FLCN/FNIP complex in multiple signaling pathways (i.e. mTOR/AMPK, TGF-β or Wnt/cadherin), and cellular processes including cell cycle, cell adhesion and migration, membrane trafficking, cilium and lysosome biogenesis, stress responses, autophagy and several others [5]. Taken together, these data point to a pivotal role of FLCN in cellular homeostasis and raise the interesting possibility that FLCN might be an important actor during fluid flow-induced autophagy and cell volume regulation in epithelial cells.

In the present study we investigated the role of FLCN in the primary cilium-dependent molecular signaling pathway that controls autophagy and cell volume in response to shear stress-induced by fluid flow in human kidney epithelial cells.

## RESULTS

### Folliculin and autophagy are increased in response to fluid flow in HK2 cells

**Figure 1:**
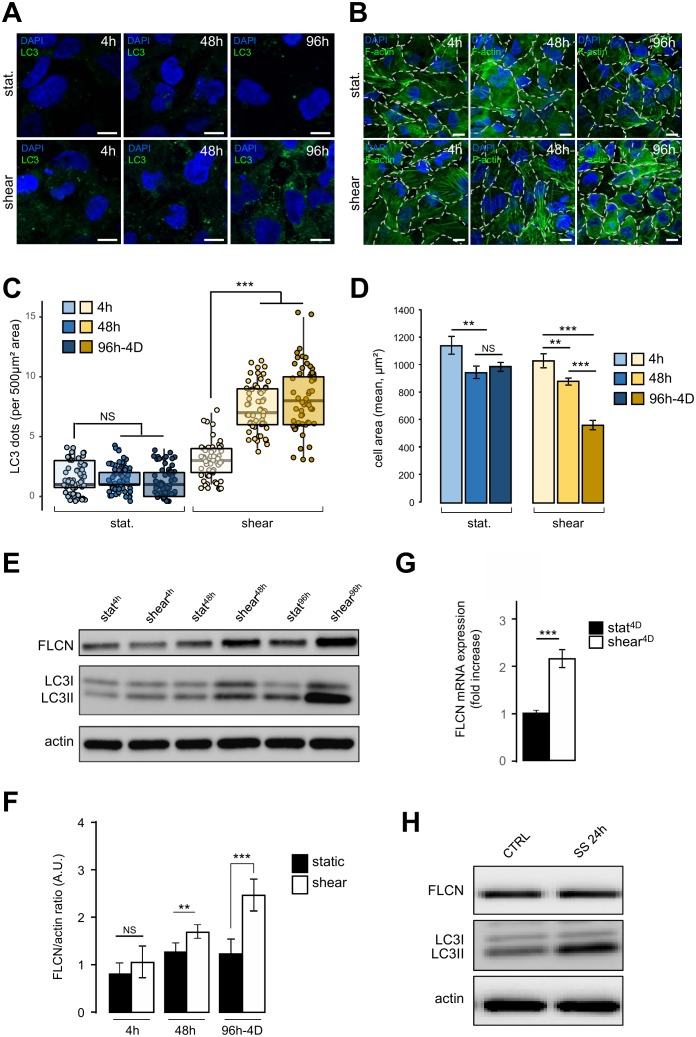
FIGURE 1: Shear stress induces autophagy, cell size decrease and FLCN expression. (A-D) HK2 cells were subjected to fluid flow from 4 h to 4 days (shear 4 h, 48 h, 96h-4D), or not (static 4 h, 48 h, 96h-4D). **(A)** After fluid flow treatment or static culture, cells were fixed, labeled with DAPI and immunostained for the autophagosome marker LC3 or stained with phalloidin to reveal F-actin and cell boarders are marked out with white dashes **(B)** and then analyzed by fluorescence microscopy. **(C)** Quantification of LC3 puncta (LC3 dots number per 500 μm² area) from experiments shown in (A). **(D)** Quantification of cell area (mean) from experiments shown in (C). **(E-F)** HK2 cells were subjected to fluid flow from 4 h to 4 days (shear 4 h, 48 h, 96h-4D), or not (static 4 h, 48 h, 96h-4D). **(E)** Representative western blot analysis of FLCN, LC3I, LC3II and actin in the indicated conditions. **(F)** Quantification of Western blot shown in (E). **(G)** HK2 cells were subjected to 4 days (shear 4D) fluid flow or not (static 4D) and FLCN mRNA expression level was determined and quantified by RT-qPCR. **(H)** HK2 cells were maintained in normal culture condition (CTRL) or subjected to a 24 h serum starvation (SS 24 h). Levels of the FLCN, LC3I and LC3II were analyzed by western blot. Scale bars in (A) and (C) = 10μm.

We have recently shown that autophagy is induced in response to shear stress in mouse KECs and in Madin-Darby canine kidney (MDCK) cells [2]. Similar results were observed in the human kidney proximal tubule HK2 cells (see **Figure 1**). To monitor dynamically the behavior of HK2 cells submitted to shear stress, we analyzed several parameters after 4 h, 48 h and 96 h-4D of fluid flow, and compared them with cells in the same culture chambers for the same periods of time, but without any fluid flow (i.e. under static conditions). As expected, a constant shear stress induced a strong autophagic response, as monitored [11] by an increase in the number of LC3 dots (**Figure 1A** and **1C**) and in the LC3 lipidation status (**Figure 1E**). Cell size was also decreased after shear stress as previously shown in KEC mouse cells [2], notably after 96 h-4D of treatment (**Figure 1B** and **1D**). Interestingly, upon shear stress the amount of FLCN protein increased in time, (**Figure 1E** and **1F**), as was the FLCN mRNA (**Figure 1G**), suggesting that shear stress induction of autophagy leads to a specific upregulation of FLCN expression at the transcriptional level. Remarkably, this was not observed in cells prone to autophagy induced by serum starvation (**Figure 1H**), suggesting that FLCN response to the autophagic machinery and its mobilization are dependent on the stress type.

### Folliculin controls the length of primary cilium in response to shear stress

**Figure 2:**
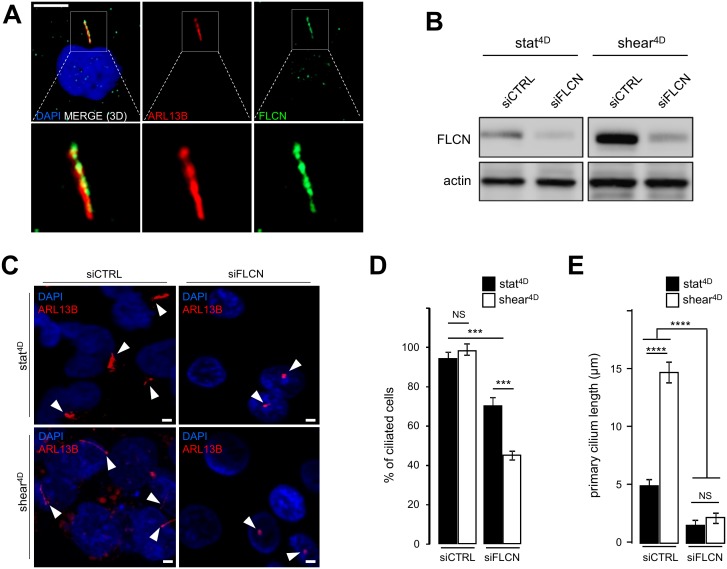
FIGURE 2: FLCN is associated with primary cilium and ciliogenesis. **(A)** HK2 cells grown for 9 6h in static conditions were fixed with methanol, labeled with DAPI, immunostained for FLCN, ARL13B (to reveal primary cilium) and then analyzed by fluorescence microscopy. **(B-E)** HK2 cells were transfected either with siRNA targeting FLCN (siFLCN) or control siRNA (siCTRL). 72 h later they were subjected to 4 days fluid flow (shear 4D) or not (static 4D). **(B)** FLCN downregulation by siRNA efficiency was verified by western blot. **(C)** Cells were fixed with methanol, labeled with DAPI, immunostained for ARL13B (to reveal primary cilium) and then analyzed by fluorescence microscopy. **(D, E)** Quantification of ciliated cells number and cilia length from experiments shown in (C). Scale bars in (A) = 10μm and (C) = 5μm.

In addition to the induction of autophagy and the decrease of the cell size, we show that fluid flow-induced shear stress promotes an increase in the primary cilium length in human HK2 cells (Supplemental Figure S1A and S1B). This is accompanied at the molecular level by the upregulation of the primary cilium regulatory protein IFT20 [3] (Supplemental Figure S1C and S1D) in response to fluid flow. We thus question the relationship between FLCN behavior and primary cilium. We show, as previously reported [6], that FLCN is present at the axoneme of primary cilium (**Figure 2A**). Upon knockdown of FLCN (**Figure 2B**), the number of ciliated cells under shear stress is affected (**Figure 2C** and **2D**) as well as the length of remaining primary cilia (**Figure 2C** and **2E**). At the molecular level, knockdown of FLCN induces a decrease of the IFT20 protein amount in a shear stress situation (Supplemental Figure S2A and S2B), highlighting the key role of FLCN in the primary cilium organelle dynamics.

### Folliculin controls autophagy-dependent cell size regulation in response to shear stress

**Figure 3:**
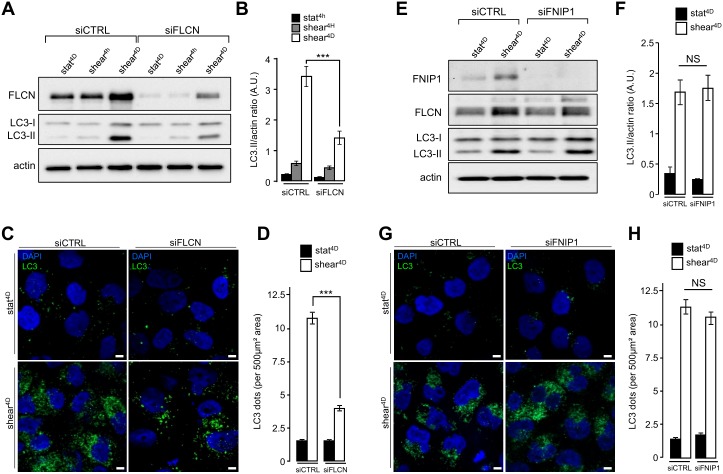
FIGURE 3: FLCN, but not FNIP1, is required for shear stress-induced autophagy. **(A-D)** HK2 cells were transfected either with siRNA targeting FLCN (siFLCN) or control siRNA (siCTRL) 72 h later they were subjected to fluid flow (shear) for the indicated times or not (static). **(A)** Levels of FLCN, LC3I and LC3II were analyzed by western blot and LC3 II/actin ratio was quantified **(B)**. **(C)** Cells were fixed, labeled with DAPI, immunostained for LC3 and then analyzed by fluorescence microscopy. **(D)** LC3 dots were quantified from experiments shown in (C). **(E-H)** HK2 cells were transfected either with siRNA targeting FNIP1 (siFNIP1) or control siRNA (siCTRL). 72 h later they were subjected to fluid flow for 4 days (shear 4D) or not (static 4D). **(E)** Levels of FNIP1, FLCN, LC3I and LC3II were analyzed by western blot and LC3 II/actin ratio was quantified **(F)**. **(G)** Cells were fixed, labeled with DAPI, immunostained for LC3 and then analyzed by fluorescence microscopy. **(H)** LC3 dots were quantified from experiments shown in **(G)**. Scale bars in (C) and (G) = 5μm.

We recently reported that primary cilium-dependent autophagy regulates KEC size decrease [2]. To better decipher the role of FLCN localized at the primary cilium in autophagy and cell size regulation, we first analyzed the shear stress-associated autophagic response in FLCN siRNA transfected cells. Lipidation of the LC3 marker in response to shear stress was strongly diminished in siFLCN compared to control cells (**Figure 3A** and **3B** and Supplemental Figure S3A and S3B), suggesting that the autophagy response induced by fluid flow is associated with FLCN, while FLCN knock-down did not affect starvation induced autophagy, as monitored by LC3 lipidation (Supplemental Figure S3C). The reduction of autophagosome biogenesis in siFLCN cells prone to shear stress was confirmed by a decrease of the total number of LC3 positive structures which presumably correspond to pre-autophagosomes, mature autophagosomes and autophagolysosomes (**Figure 3C** and **3D**). However, downregulation of the folliculin-interacting protein FNIP1 [12], has no effect either on LC3 lipidation (**Figure 3E** and **3F**) or on the number of LC3 positive structures (**Figure 3G** and **3H**) in response to fluid flow, suggesting that FLCN function in shear stress associated autophagy is independent of its FNIP1 partner.

**Figure 4:**
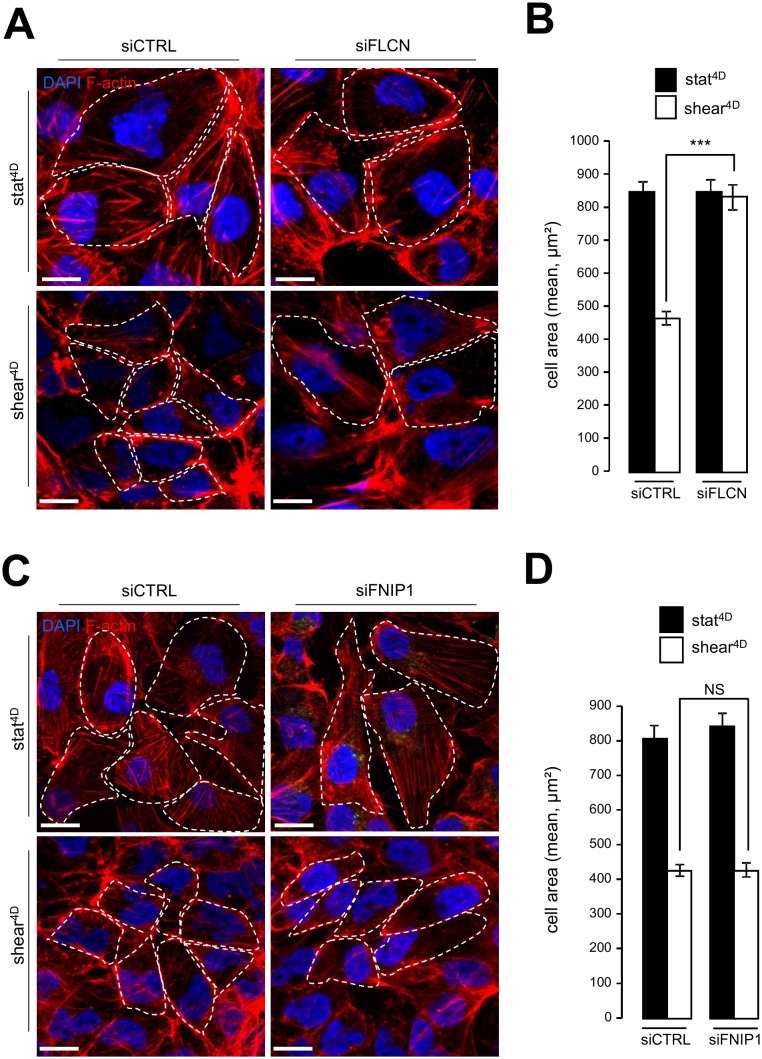
FIGURE 4: FLCN, but not FNIP1, is required for shear stressinduced cell size regulation. **(A-B)** HK2 cells were transfected either with siRNA targeting FLCN (siFLCN) or control siRNA (siCTRL) 72 h later they were subjected to fluid flow for 4 days (shear 4D) or not (static 4D). **(A)** Cells were fixed, labeled with DAPI and phalloidin to reveal F-actin and cell boarder and then analyzed by fluorescence microscopy. **(B)** Cells areas were quantified from experiments shown in (A). **(C-D)** HK2 cells were transfected with siRNA targeting FNIP (siF-NIP1) or control siRNA (siCTRL)). The experiments were performed and quantified as in (A-B). Scale bars in (A) and (C) = 10μm.

Likewise, knocking down FLCN impairs cell size decrease in response to fluid flow (**Figure 4A**, **4B**), while the knockdown of FNIP1 has no effect on fluid flow-induced cell size decrease (**Figure 4C**, **4D**). In line with cell size regulation and autophagy interplay induced by mechanical stress, we observed defaults in the mTORC1 pathway activation in FLCN knockdown cells, since phosphorylation of TSC2, known to inactivate the mTOR signaling sequence, was strongly diminished in siFLCN cells prone to shear stress (Supplemental Figure S4A and S4B). Moreover, the phosphorylation status of the S6 protein, a downstream target in the mTOR signaling pathway, was strongly increased in FLCN knock-down cells prone to fluid flow compared to control cells (Supplemental Figure S4A and S4C).

**Figure 5:**
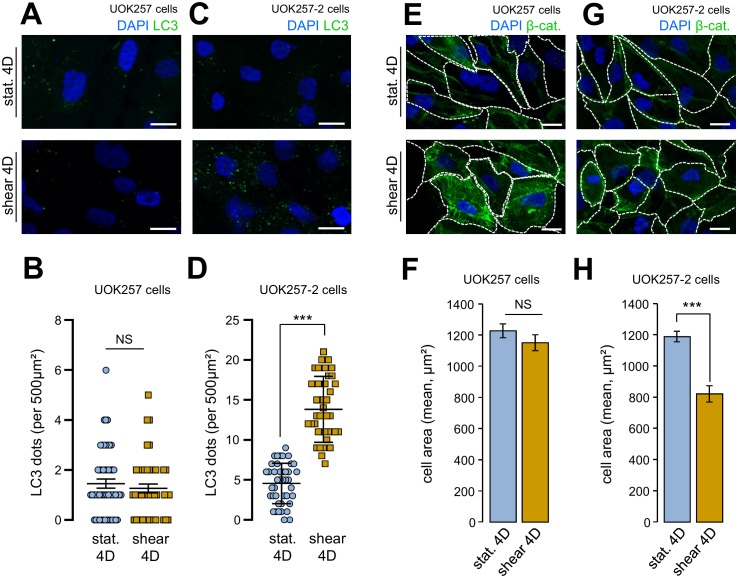
FIGURE 5: Shear-stress-induced autophagy and cell size regulation are abolished in FLCN-null cells. UOK 257 FLCN-null cells and UOK 257-2 FLCN restored cells were cultured on microslides and then subjected to fluid flow for 4 days (shear) or not (static 4D). Cells were fixed, labeled with DAPI, immunostained for LC3 **(A, C)** or labeled with DAPI and immunostained for β-catenin to reveal cell boarder **(E, G)** and then analyzed by fluorescence microscopy. **(B, D)** LC3 dots were quantified from experiments shown in (A) (UOK 257cells) and (C) (UOK 257-2 cells). **(F, H)** cells areas were quantified from experiments shown in (E) (UOK 257cells) and (G) (UOK 257-2 cells). Scale bars in (A, C, E and F) = 10μm.

We confirmed to importance of FLCN in cell size adaptation and autophagy in response to fluid flow using a Birt–Hogg–Dubé derived FLCN-null cells (UOK 257cells) and FLCN restored companion cells (UOK 257-2 cells) [12, 13]. Indeed, UOK 257cells prone to shear stress did neither display any autophagy change, as monitored by the number of LC3 positive structures (**Figure 5A** and **5B**), nor cell size adaptation to the fluid flow treatment (**Figure 5E** and **5F**). Importantly, UOK 257-2 cells, in which the FLCN expression is restored, display identical features than control HK2 cells (**Figure 1**) and other kidney cells [2] regarding autophagic machinery mobilization under shear stress (**Figure 5C** and **5D**) and cell size adaptation (**Figure 5G** and **5H**). These data strengthened the hypothesis that FLCN is central for autophagy induced by mechanical stress in kidney epithelial cells.

**Figure 6:**
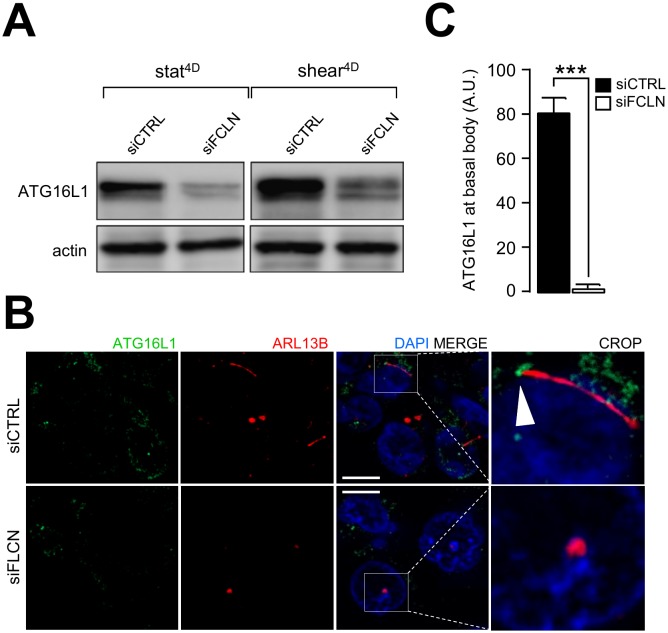
FIGURE 6: Shear-stress dependent recruitment of ATG16L1 to primary cilium is impaired in FLCN knockdown cells. HK2 cells were transfected with control siRNA (siCTRL) or with siRNA targeting FLCN (siFLCN). 72 h later, they were subjected to fluid flow for 4 days (shear 4D) or not (static 4D). **(A)** ATG16L1 and actin levels were analyzed by western blot. **(B)** Cells prone to fluid flow were fixed with methanol, labeled with DAPI, immunostained for ATG16L1 and ARL13B and then analyzed by fluorescence microscopy. Arrowhead indicates presence of ATG16L1 at the basal body. **(C)** ATG16L1 positive structures at basal body were quantified from experiments shown in (B). Scale bar = 10μm.

A hallmark of primary cilium-dependent autophagy is the recruitment of components of the autophagy machinery at the primary cilium [14]. We have previously shown that ATG16L1 is recruited at the basal body in response to shear stress [2]. Interestingly, knocking down FLCN leads to ATG16L1 destabilization (**Figure 6A**) and impairs its recruitment at the basal body of primary cilium (**Figure 6B** and **6C**), emphasizing the role of FLCN in setting up autophagy upon fluid flow.

Overall these results show that FLCN is a key player in the regulation of fluid flow-induced autophagy and cell size regulation.

### Folliculin is upstream of AMPK and LKB1 in the primary cilium signaling cascade

**Figure 7:**
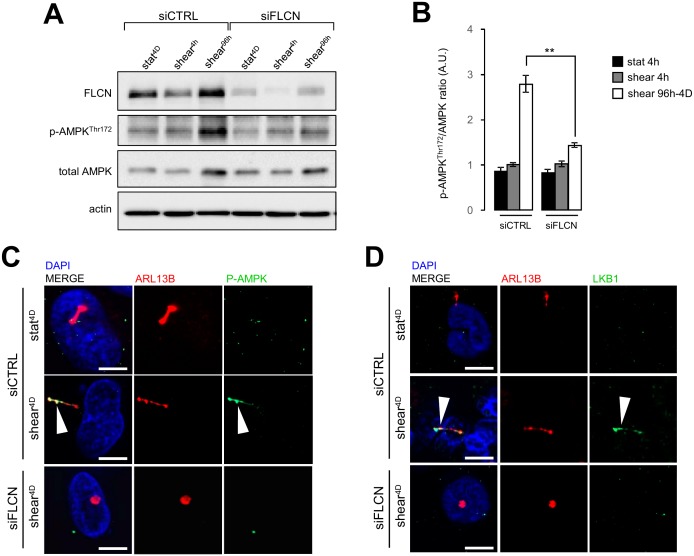
FIGURE 7: FLCN is associated with phospho-AMPK and LKBI mobilization during shear stress. HK2 cells were transfected with control siRNA (siCTRL) or with siRNA targeting FLCN (siFLCN). 72 h later, they were subjected (shear) or not (static) to fluid flow for the indicated times. **(A)** Phospho-AMPK (Thr172), total AMPK, FLCN and actin levels were analyzed by western blot and quantified **(B**) in the indicated conditions (A). **(C, D)** Cells prone to fluid flow (shear 4D) or not (static 4D) were fixed with methanol, labeled with DAPI, immunostained for ARL13B, phospho-AMPK **(C)**, LKB1 **(D)**, ARL13B, and then analyzed by fluorescence microscopy. Arrowheads indicate presence of phospho-AMPK (P-AMPK) or LKB1 at primary cilium. Scale bars in (C) and (D) = 10μm.

Stress sensing signalization induced by the bending of primary cilium in epithelial cells has been associated with local recruitment of LKB1 and AMPK at the primary cilium and local phosphorylation of the latter [15, 16]. This signaling cascade controls primary cilium-dependent autophagy [2] and FLCN has been shown to contribute to the recruitment of LKB1 on the primary cilium to regulate activation of AMPK and mTORC1 during shear stress [6]. In our experimental system we observe as well that under shear stress, AMPK phosphorylation depends on the presence of FLCN (**Figure 7A** and **7B**), so does its recruitment to the primary cilium (**Figure 7C**). Moreover, knocking down FLCN prevents LKB1 targeting to the primary cilium in cells prone to shear stress (**Figure 7D**). These findings suggest that FLCN orchestrates the AMPK/LKB1 signaling machinery at the primary cilium to regulate autophagy and cell size in kidney epithelial cells.

## DISCUSSION

In the present work we identify FLCN as a component of the primary cilium signaling cascade that senses fluid flow to regulate autophagy and cell size in kidney epithelial cells. FLCN is upregulated upon shear stress, and it functions in modulating autophagic-dependent cellular size decrease. Ciliary length is also dependent on the levels of FLCN. These findings are in line with the observation that FLCN level affects the timing of ciliogenesis in HK2 cells [5]. Interestingly, autophagy has been shown to influence the ciliary length as well [14, 17]; whether the implication of FLCN in the control of cilia length is related to its role in autophagy and/or in its regulation of mTOR activity [17] is an open question.

In accordance with the observations of Zhong *et al*. [6], our study shows that FLCN acts upstream of AMPK and mTOR to control autophagy in response to shear stress. FLCN contributes to the recruitment of LKB1 to the primary cilium, which is important for the activation of AMPK [6]. FLNC-associated proteins FNIP1 and FNIP2 have been shown to be engaged in a complex with AMPK, and the loss of FNIP1 stimulates AMPK and increases autophagy in B cells [18]. However, in our experimental setting, knockdown of FNIP1 does neither impact autophagy nor fluid flow-dependent cell size. Thus, although the implication of FLCN and its associated proteins FNIP1 and 2 in autophagy has been studied in various settings [7, 8, 18], it is difficult to summarize it in a unified picture. It is most probably dependent on the cell type, the stimuli of autophagy and their subcellular localization (e.g., primary cilium *vs* lysosomal membrane).

Our study adds a new component to the signaling cascade emanating from the primary cilium in response to fluid flow to regulate autophagy. However, the relation between this signaling cascade and the mechanosensor present in the primary cilium associated membrane remains to be identified. The complex formed by polycystin 1 (PC1) and polycystin 2 (PC2) functions as a calcium channel at the primary cilium [19, 20]; in this complex PC1 is a mechanosensor [21]. Our previous studies have shown that PC2 is not involved in the autophagy cascade leading to cell size regulation in response to fluid flow [2]. However, it remains a possibility that PC1 is upstream of FLCN to regulate this cascade and cell size in kidney epithelial cells. Further experiments should challenge this hypothesis.

In conclusion we show that FLCN localized at the primary cilium regulates autophagy and cell size in kidney epithelial cells in response to shear stress induced by fluid flow. Our work is in line with the fact that autophagy is inhibited in clear cell tumors from a BHD patient [7]. Further studies should address whether this physiological response is altered in BHD patients.

## MATERIALS AND METHODS

### Cell culture and siRNA transfection

Human kidney HK2 cells (from ATCC) and Birt-Hogg-Dube syndrome associated FLCN-null human kidney UOK 257 cells (as well as FLCN-restored UOK257-2 cells) (from Dr Laura Schmidt (National Cancer Institute, NIH, Bethesda)) were cultured in Dulbecco's Modified Eagle Medium (DMEM), supplemented with 10% FCS at 37°C and 5% CO_2_.For the starvation experiments, cells were cultured in Earle's balanced salt solution (EBSS) for the indicated times. siRNA transfections were performed using Lipofectamine RNAi Max (Invitrogen, Life Technologies) according to the manufacturer's instructions. Two siRNA oligomers were used for each target at a final concentration of 20 nM. All siRNAs were purchased from Qiagen and the references are as follows: Control (SI1027281); FLCN (SI05121417 and SI00387660); FNIP1 (a): (SI03222611 and SI05001766).

### Shear stress induction

HK2 cells were seeded (2.25×10^5^ in 150 μl of culture medium) into a microslide “I0.6 Luer” chamber (channel dimensions: 50 x 5 x 0.4 mm, Ibidi) and cultured for 96 h to allow proper polarization and epithelial differentiation. The microslides were connected to a fluid flow system which contains an airpressure pump and a two-way switch valve that pumps the culture medium unidirectionally between two reservoirs through the flow chamber at a rate corresponding to a shear stress of 1 dyn/cm². The control cells (static) were set up in the same microslides Luer chambers and maintained in culture as long as the flow-subjected cells.

### Protein extraction, immunoblot analysis and antibodies

Cells in microslides were washed twice with ice-cold PBS and lysed on ice with 150 μl of 1X Laemmli buffer (60 mM Tris-HCl pH=6.8, 2% SDS, 10% Glycerol, bromophenol blue, supplemented with 100 mM DTT) for 30 min. Samples were boiled for 10 min at 95°C, separated by SDS/PAGE and then transferred onto Nitrocellulose membranes. Western blot analysis was performed with specific antibodies and the antigen–antibody complexes were visualized by chemiluminescence (Immobilon Western, Merck Millipore). The following antibodies were used in immunoblotting: rabbit-anti LC3 (Sigma, Cat#L7543); rabbit-anti-FLCN (Cell signaling, Cat#3697); rabbit-anti-FNIP1 (Abcam, Cat#ab134969); rabbit-anti-AMPK (Cell signaling, Cat#2532S); rabbit-anti-p-AMPK (T172) (Cell signaling, Cat#2535); mouse-anti-actin (Millipore, Cat#1501); rabbit-anti-ATG16L1 (MBL, Cat#PM040); rabbit-anti-IFT20 (Proteintech, Cat#13615-1-AP); rabbit-anti β-catenin (Cell signaling, Cat#8480); rabbit-anti-LKB1 (Cell signaling, Cat#3050); rabbit-anti-S6 ribosomal protein (Cell signaling, Cat#2217); rabbit-anti-p-S6 ribosomal protein (S240/244) (Cell signaling, Cat#2215); rabbit-anti-Tuberin/TSC2 (Cell signaling, Cat#4308); rabbit-anti-p-Tuberin/TSC2 (T1462) (Cell signaling, Cat#3617). Secondary HRP conjugate anti-rabbit IgG (GE Healthcare) and HRP conjugate anti-mouse IgG (Bio-Rad).

### Immunofluorescence and microscopy

Cells were fixed either with 4% paraformaldehyde (PFA) for 20 min or with cold methanol for 5 min at -20°C for proper axoneme proteins detection [22]. They were then washed and incubated for 30 min in blocking buffer (10% FCS in PBS) followed by incubation with primary antibodies diluted in blocking buffer supplemented with 0.05% saponin for 1 h at room temperature or overnight at 4°C. Cells were washed 3 times, and then incubated for 1 h with fluorescent Alexa-Fluor secondary antibodies. After washing, 150 μl of DAPI-Fluoromount were added into the Luer chamber (Southern Biotech). Images were acquired with a Zeiss Apotome.2 fluorescence microscope equipped with a 63x oil immersion fluorescence objective. Number of ciliated cells and length of cilia were quantified using Zen Software (Zeiss) or Imaris Software (Bitplane). The following antibodies were used for immunofluo-rescence: mouse-anti-LC3B (MBL, Cat# M152-3); rabbit-anti-FLCN (Cell signaling, Cat#3697); mouse-anti-ARL13B (C-5) (Santa Cruz, Cat#515784); rabbit-anti-ATG16 (MBL, Cat#PM040); rabbit-anti IFT20 (Proteintech, Cat#13615-1-AP); rabbit-anti-p-AMPK (T172) (Cell signaling, Cat#2535); Phalloidin (Cat# A34055); Alexa Fluor-conjugated secondary anti-bodies (donkey anti-mouse IgG and donkey anti-Rabbit IgG, Life Technologies).

### Real Time Quantitative PCR

RNA was extracted from cells using the NucleoSpin RNA kit (Macherey-Nagel). Reverse transcriptase PCR and qRT-PCR were performed using “Power Sybr green cells to CT” kit (Thermo Fisher Scientific) according to manufacturer's instructions. Actin was used as reference gene and relative quantification was calculated using the ΔΔCT method. Primers sequences are as followed:

Flcn-forward: 5′-TTCACGCCATTCCTACACCAGA-3′;

Flcn-reverse 5′-GCCCACAGGTTGTCATCACTTG-3′

Actin-forward 5′-GGCCAACCGTGAAAAGATGA-3′;

Actin-reverse 5′-ACCAGAGGCATACAGGGACAG-3′

### Statistical analysis

Data are presented as means ± SD or SEM. Statistical analyses were performed by unpaired, two-tailed Student's t-test, using GraphPad Prism7 (*p < 0.005, **p < 0.001, and ***p < 0.0001). Images showing Western blotting or immunofluorescence analysis are representative of three independent experiments unless stated otherwise.

## AUTHOR CONTRIBUTIONS

N.Z., A.B. and JB performed most of the biological experiments and analyses, N.D. and J.B. analyzed parts of the cell biological assays, P.C. supervised the project and wrote the paper and E.M. contributes to imaging experiments, analyzed parts of the cell biological assays, supervised the project and wrote the paper.

## SUPPLEMENTAL MATERIAL

Click here for supplemental data file.

All supplemental data for this article are available online at http://www.cell-stress.com/researcharticles/2019a-zemirli-cell-stress/.

## Abbreviations:

BHD: Birt-Hogg-Dubé,
FLCN: folliculin,
FNIP: folliculin-interacting protein,
KEC: kidney epithelial cell.
