# Supplementary material for: The primary cilium protein folliculin is part of the autophagy signaling pathway to regulate epithelial cell size in response to fluid flow
Source: Cell Stress. 2019 Feb 25;3(3):100–9. doi: 10.15698/cst2019.03.180 (PMC6551741; doi:10.15698/cst2019.03.180)
Supplement: Supplementary file 1 [file ces-03-100-s01.pdf]

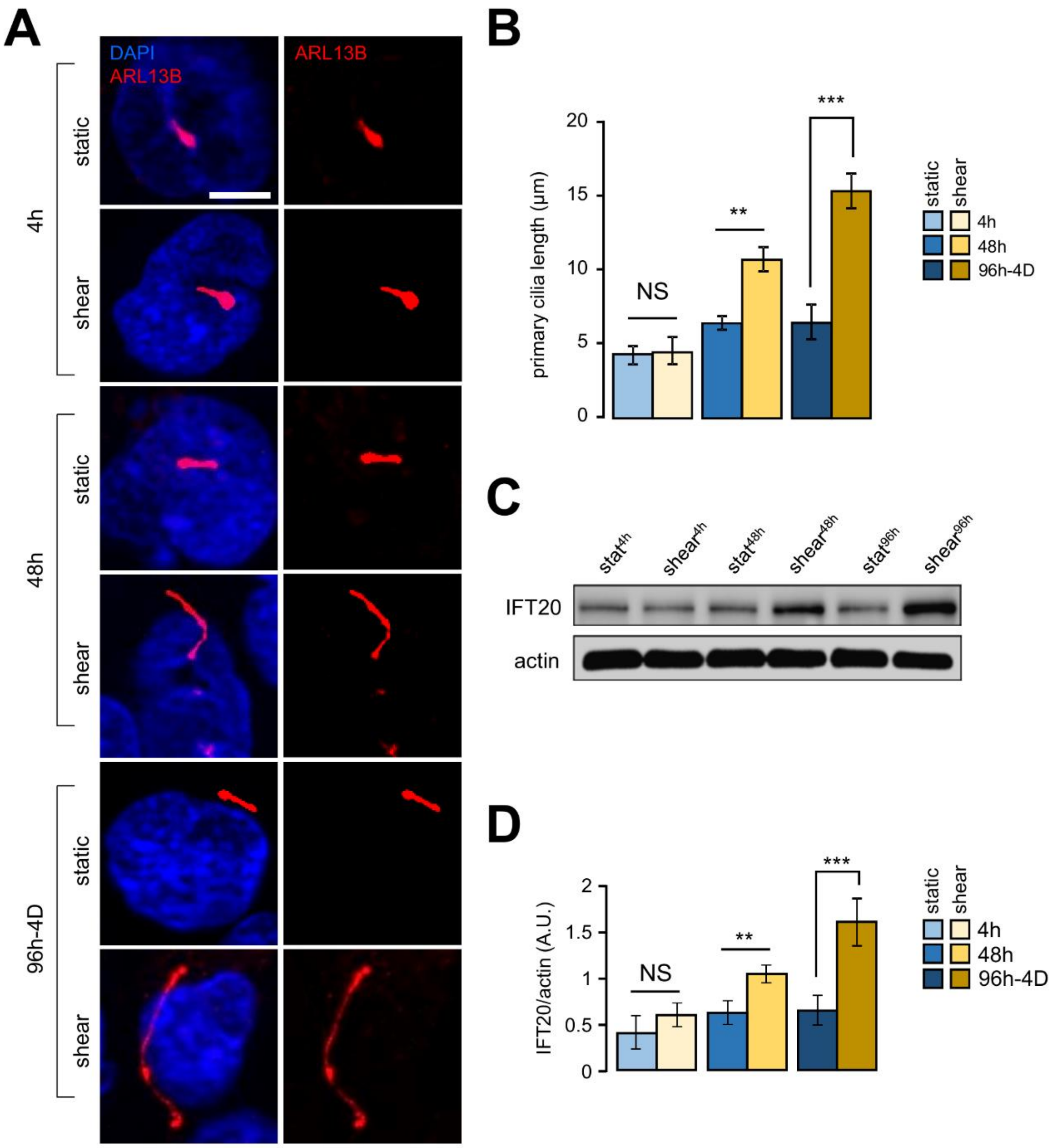

Supplementary Figure S1: Primary cilium and shear stress. (A-D) HK2 cells were subjected to fluid flow from 4h to 4 days (shear 4h, 48h, 96h-4D), or not (static 4h, 48h, 96h-4D). **(A)** Cells were fixed with methanol, labeled with DAPI and ARL13B antibody to stain the primary cilium. **(B)** Cilia length was quantified from experiments shown in (A). **(C)** Levels of the ciliary protein IFT20 were analyzed by western blot and IFT20/actin ratio was quantified **(D)**. Scale bar in (A) = 10µm.

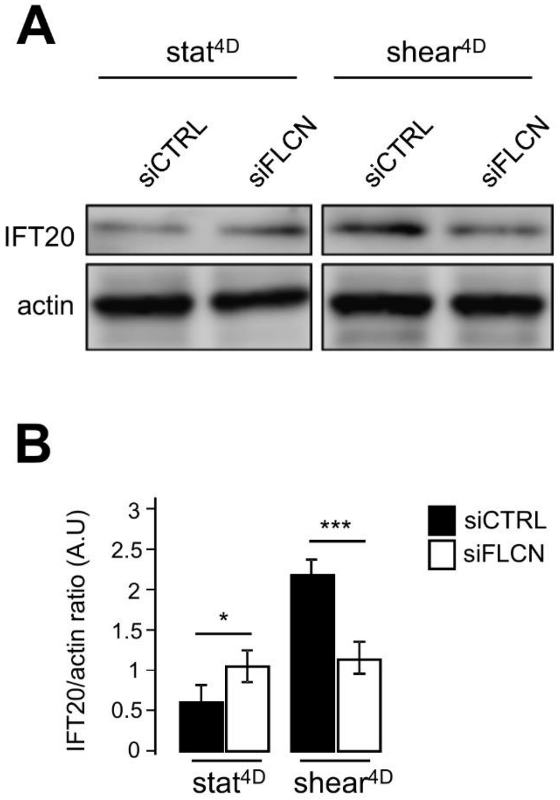

Supplementary Figure S2: FLCN knockdown affects IFT20 protein stability. HK2 cells were transfected with control siRNA (siCTRL) or with siRNA targeting FLCN (siFLCN). 72 h later, they were subjected to fluid flow for 4 days (shear 4D) or not (static 4D). **(A)** IFT20 and actin levels were analyzed by western blot and IFT20/actin ratio was quantified **(B)**.

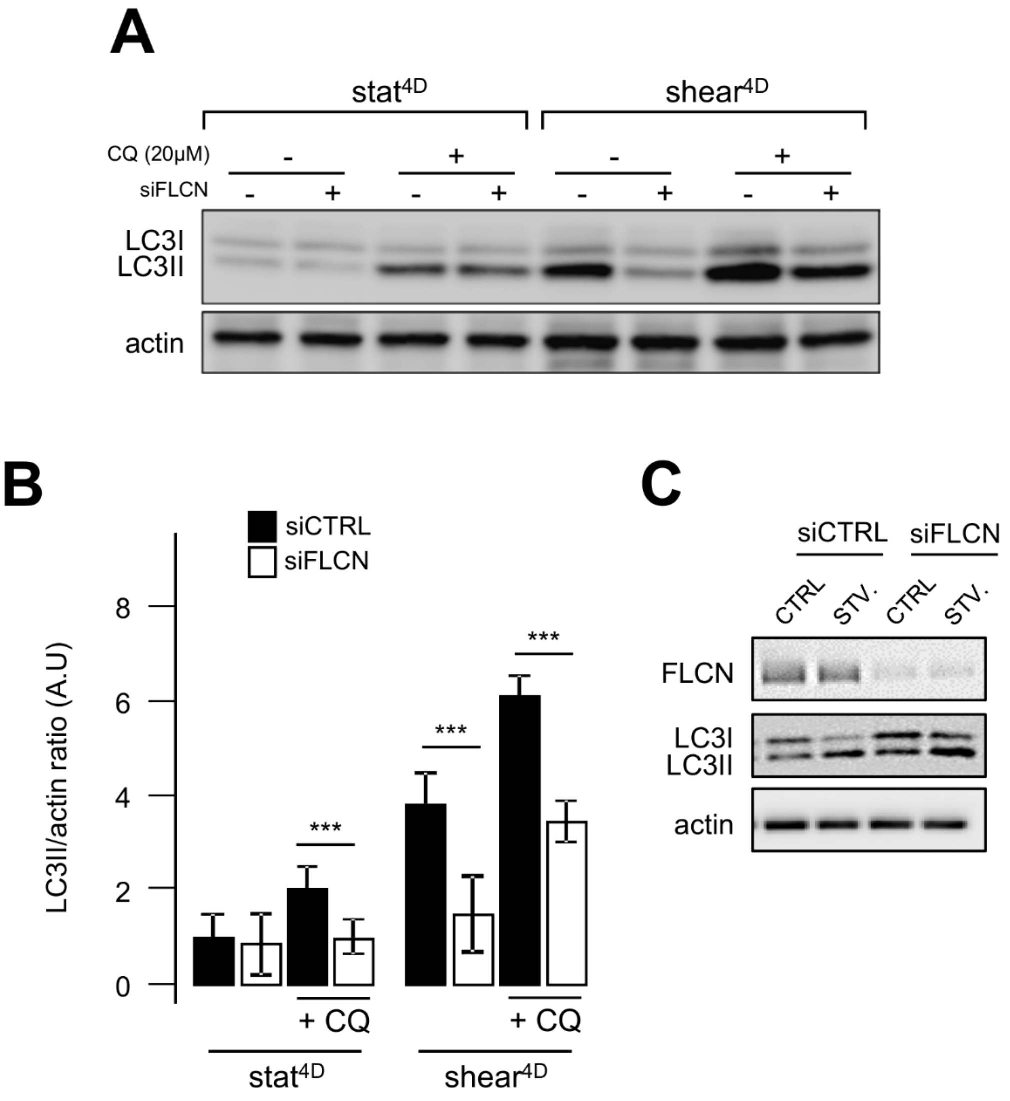

Supplementary Figure S3: FLCN and autophagy dynamics. HK2 cells were transfected with control siRNA (siCTRL) or with siRNA targeting FLCN (siFLCN). 72 h later, they were subjected to fluid flow for 4 days (shear 4D) or not (static 4D). Chloroquine (20 µM) was added (+) or not (-) during the 24 last hours of shear stress to block autophagolysosomal maturation. **(A)** LC3 and actin levels were analyzed by western blot and LC3 II/actin ratio was quantified **(B)**. **(C)** SiCTRL and siFLCN cells were grown under control condition (CTRL) or upon complete starvation condition for 24 h (STV.) and FLCN, LC3 and actin levels were analyzed by western blot.

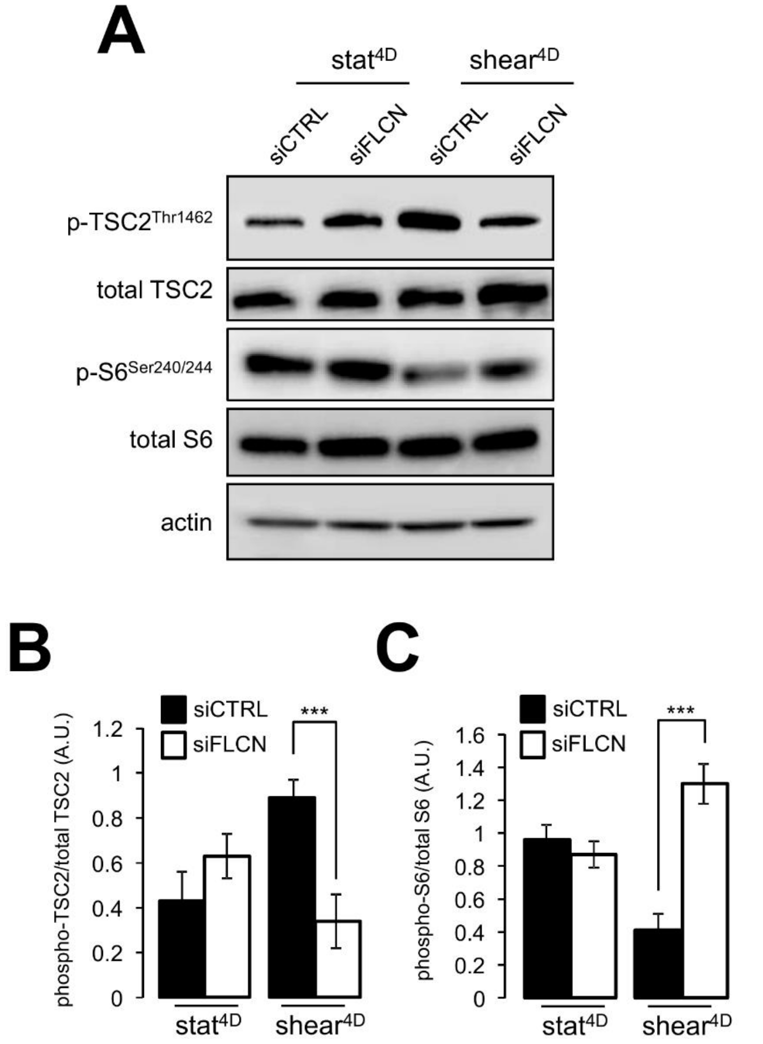

Supplementary Figure S4: FLCN knockdown affects mTOR signaling pathway in response to fluid flow. HK2 cells were transfected with control siRNA (siCTRL) or with siRNA targeting FLCN (siFLCN). 72 h later, they were subjected to fluid flow for 4 days (shear 4D) or not (static 4D). **(A)** phospho-TSC2, total TSC2, phospho-S6, total S6 and actin levels were analyzed by western blot and normalized phospho-TSC2/total TSC2 ratio and normalized phospho-S6/total S6 ratio were quantified **(B, C)**.
